# Factors Affecting Delayed Hospital Arrival of Patients with Acute Myocardial Infarction in Kinmen

**DOI:** 10.3390/ijerph19031323

**Published:** 2022-01-25

**Authors:** Yu-Han Huang, Chorng-Kuang How, Ching-Sung Ho

**Affiliations:** 1 Cardiovascular Care Center, Kinmen Hospital, Ministry of Health and Welfare, Kinmen 89142, Taiwan; cindy7303067676@kmhp.mohw.gov.tw; 2 Emergency Department, Taipei Veterans General Hospital, Taipei 11217, Taiwan; ckhow@vghtpe.gov.tw; 3 Department of Emergency Medicine, School of Medicine, National Yang Ming Chiao Tung University, Taipei 11221, Taiwan; 4 Department of Long Term Care, National Quemoy University, 1 University RD. Jinning Township, Kinmen 89250, Taiwan

**Keywords:** acute myocardial infarction, health visit delay, Killip Class, Kinmen

## Abstract

This study explores factors related to delayed emergency medical treatment for acute myocardial infarction patients on Kinmen Island. A cross-sectional study was performed in the only hospital in Kinmen Island. The study group consisted of 116 patients diagnosed with acute myocardial infarction (AMI, ICD-10 codes: I21.9) from November 2015 to May 2019. The binary logistic regression analyses were performed for the inferential statistical analysis. The mean age of the study group was 63.0 ± 14.5 years, 39.7% of the patients arrived at the emergency medicine longer than 6 h after the onset of symptoms. The related factors for delayed arrival the hospital emergency medicine department were female sex, age over 65 years, less than nine years’ education, and Killip Class, but only Killip Class reached the significant difference of statistics (OR = 3.616, 95% C.I. = 1.574 to 8.310, *p* = 0.002), and patients with delayed arrival times (>6 h) were found to have a higher percentage of Killip Class ≥ II. Therefore, it is essential to remind the physicians to proceed with risk stratification for acute coronary syndrome patients. In addition, health authorities should provide effective programs to increase awareness of the symptoms and timely treatment of acute myocardial infraction to the general public, especially the elderly.

## 1. Introduction

Heart disease is a major health problem worldwide and is currently the leading cause of death in the USA [1]. There are more than 1000 deaths caused by cardiovascular diseases (CVD) daily, with 2.2 million hospitalizations and 415,480 deaths in 2016 [2]. In 2017, about 17.8 million deaths were attributed to CVD globally, an increase of 21.1% from 2007 [3]. Heart disease has been the second leading cause of death in Taiwan since 2007 [4]. There were 20,644 deaths due to heart disease in 2016 [5].

Myocardial infarction is the main contributor to the high incidence of heart failure [6,7]. Acute myocardial infarction (AMI) is defined as cardiomyocyte necrosis in a clinical setting consistent with acute myocardial ischemia, the detection of a rise and/or fall of cTn values with at least one value above the 99th percentile URL and at least one of the five indications: (1) symptoms of myocardial ischemia; (2) new ischemic electrocardiogram (ECG) changes; (3) development of pathological Q waves; (4) imaging evidence of new loss of viable myocardium or new regional wall motion abnormality in a pattern consistent with an ischemic etiology; (5) identification of a coronary thrombus by angiography or autopsy [8].

ST-elevation myocardial infarction (STEMI) is defined as ST elevation or left bundle branch block on the first or subsequent 12-lead electrocardiogram (ECG). In contrast, patients without ST-segment elevation at presentation are usually designated as having a non-ST-segment elevation myocardial infarction (NSTEMI) [9]. In the USA, there were six times as many patients with STEMI versus NSTEMI in 1990, but by 2000, the proportions had equalized, and subsequently, the proportion of NSTEMI exceeded that of STEMI [10]. Similarly, NSTEMI to STEMI incidence ratio increased from 1.93 in 2009 to 2.47 in 2015 in Taiwan [11]. This may be due to the increased use of troponin as a sensitive biomarker for diagnosing AMI, an aging population, the growing prevalence of antecedent coronary revascularization, the increased use of aspirin, and improved health system and medical therapy [10].

The Killip classification is a common clinical tool for cardiovascular assessment and risk stratification. According to Killip classification, patients are categorized into four classes during clinical examination. Patients in Class I have no evidence of heart failure (HF), patients in Class II had indications along with mild to moderate HF, patients in Class III had obvious pulmonary edema, and patients in Class IV were in cardiogenic shock [12]. Patients with higher Killip Class were found to have more severe angiographic coronary artery disease, higher occurrence of ventricular failure, and greater myocardial infarctions [13].

For patients who receive prompt care, reperfusion therapy including surgical or pharmacologic treatments can modify the progression of a heart attack and limit heart damage and subsequent morbidity and mortality [14,15]. The shorter the interval, the better the outcome, so patients should arrive at the hospital emergency room as soon as possible to maximize the chances of survival.

Delay in seeking medical treatment after symptoms begin may be due to misunderstanding the urgency of signs and symptoms, or psychological denial [16]. Numerous studies have found socio-demographic and clinical issues related to delayed treatment after the onset of a heart condition, and the elderly, females and those with a history of diabetes tend to have a longer delay in seeking medical treatment than the others [17,18,19,20]. Internationally, the median prehospital delay for AMI patients ranged from 2 to 6.5 h [21], and females had a longer delay in obtaining hospital treatment than males (median: 270 min versus 240 min) [22]. Therefore, it is important to clarify the gender difference between the onset of disease symptoms and the patient’s arrival at a hospital emergency department.

Kinmen is a small island of 150 square kilometers, located near the major Chinese city of Xiamen, and administered as part of Taiwan. It has a permanent resident population of nearly 60,000 people and only one public district hospital (Kinmen Hospital of the Ministry of Health and Welfare), with 300 beds, which is the major center for medical care. The standardized mortality rate is 314.5 per 100,000 residents for all causes and heart disease is the second leading cause of death, as in Taiwan overall [23]. Although Kinmen Hospital is the only medical facility caring for HF patients and provides percutaneous coronary intervention (PCI) 24/7, patients diagnosed as too severe to be treated are transferred to major medical centers in Taiwan by plane or helicopter. Therefore, it is important to propose effective intervention strategies to help patients detect physiological signs of acute myocardial infarction and seek medical care promptly.

This study explored factors related to a delayed response to AMI in Kinmen and proposes suggestions for developing interventions to reduce the time from onset of symptoms to obtaining proper medical care.

## 2. Materials and Methods

A hospital-based cross-sectional study was conducted in Kinmen from January 2020 to June 2020 at the Kinmen hospital of the Ministry of Health and Welfare. The study sample consisted of 116 patients, aged 37 to 100 years, diagnosed with acute myocardial infarction (AMI, ICD-10 codes: I21.9) at the emergency department. This follows the definition of the European Society of Cardiology and the American College of Cardiology (ESC/ACC): The existence of at least two of the following three features: (1) Chest pain lasting more than 20 min, (2) ST-elevation of at least 2 mm in two or more contiguous leads with successive evolution of the electrocardiogram, and (3) elevated cardiac marker (CK-MB) or positive troponin I or T [24]. All patients in the study group were admitted between 20 November 2015, and 31 May 2019. According to ESC guidelines, two kinds of time intervals are mainly important: (1) the time delay between the onset of symptoms and the first medical contact (FMC) and (2) the time delay between FMC and the beginning of reperfusion [25]. Early (≤6 h) treatment of PCI on the artery responsible for the myocardial infarction is a significant determinant factor of survival beyond the hospital phase of ST-elevation myocardial infarction [26]. The emergency medical service travel time is usually less than 30 min in Kinmen. Late arrival was defined as a time interval from the onset of symptoms to arrival at the hospital emergency room (ER) greater than six hours.

### 2.1. Statistical Analysis

Frequency analyses were conducted to assess AMI patients’ late arrival distribution and classify the associated factors of late arrival. The distribution of late arrival was obtained for the arrival time from onset of symptoms to arrival at the ER. To assess significant issues related to patients’ arrival levels, the Chi-Square test and binary logistic regressions were performed for inferential statistical analysis. The SPSS software package (version 18.0 was used for statistical analyses. The significance level for the Alpha value was set at 0.05).

### 2.2. Ethics Approval

The study protocol was approved by the Medical Ethics Committee of National Cheng Kung University (IRB no. 108-331). The institutional review board of the Medical Ethics Committee of National Cheng Kung University approved this study without requiring written informed consent from the patients under study.

## 3. Results

The study included 116 patients with a mean age of 63.0 ± 14.53 years, with 69 (59.5%) above 65 years of age. There were 64 (55.2%) with no more than nine years’ education, most (94.8%) were married, and 101 (87.1%) were living with their family. Furthermore, 91.3% of the patients arrived at the ER by ambulance, 69 (59.5%) were diagnosed with ST-elevation myocardial infarction (STEMI), 60 (51.7%) were of Killip Class I, and only two were on IV. Comparing the chronic disease status, 60 (51.7%) had hypertension, 33.6% and 22.4% had diabetes mellitus and hyperlipidemia, respectively. 70 (60.3%) were found to have reached the ER equal to or less than 6 h after the onset of symptoms. The samples’ basic sociodemographic and clinical characteristics are shown in Table 1.

Differences in the time to ER distribution among the AMI patients by the X^2^ tests are shown in Table 2. There were various variables such as marital status, living with family status, and the means of travel to the ER, but most of the cases were concentrated in a single category. As such, inferential statistics analysis was not used Since there were only 27 patients with Killip scores three or higher, cases whose Killip Class was ≥II were combined as the high score group for the inferential analysis. Variables that reached significance included gender (*p* = 0.011), age (*p* = 0.004), educational level (*p* = 0.025), and Killip Class (*p* = 0.001). Comparing gender and time to ER, 65.0% of females showed > 6 h to ER, compared to 34.4% of males. A higher prevalence of time to ER > 6 h was related to higher age, with 53.3% of the patients older than 65 having time to ER longer than 6 h, while 29.0% of those 64 or younger were in this bracket. There was a higher prevalence of time to ER > 6 h in patients with less education. Among the patients with time to ER > 6 h, 57.0% had ≤ nine years of education, compared to 27.1% with 10 or more years of schooling. In the group of time to ER > 6 h, 57.1% had Killip Class of I, while 42.9% were equal or higher than II. The AMI patients diagnosed as STEMI were found to have a higher prevalence of time to ER > 6 h than NSTEMI (48.9% vs. 33.3%), but AMI type and chronic disease status, such as hypertension, diabetes mellitus, and hyperlipidemia, did not reach statistically significant difference (*p* > 0.05).

Table 3 shows results from the binary logistic regression analyses, indicating that the difference of time to ER among genders, age groups, and educational levels did not reach significance. This result illustrates that the Killip Class is a correlation factor for time to ER distribution. Patients with delayed arrival times (>6 h) were found to have a higher percentage of Killip Class ≥ II. (OR = 3.616, 95% C.I. = 1.574 to 8.310, *p* = 0.002).

The prevalence of time to ER > 6 h for female patients was higher than for males (OR = 1.084) However, the distribution of time to ER among gender, age group, and educational level did not significantly differ.

The limited case numbers may lead to underpowered results. Table 3a,b indicate the results of two independent variables analyzed in the binary logistic regression analyses. Killip Class reached a significant difference in both models (OR = 3.936, 95% C.I. = 1.736 to 8.823, *p* = 0.001 and OR = 4.214, 95% C.I. = 1.835 to 9.266, *p* = 0.001), respectively. Higher age (≥65) having time to ER longer than 6 h than those 64 or younger (OR = 2.602, 95% C.I. = 1.149 to 5.895, *p* = 0.022) and educational levels did not reach statistically significant difference.

## 4. Discussion

To the best of our knowledge, this is the first study to analyze the clinical and sociodemographic factors for the time to ER of AMI patients in Kinmen Island. This study finds that almost 40% of the AMI patients were late for arrival to the ER. This is higher than prior studies reported that 25% to 30% of AMI patients arrive at the hospital >6 h after symptom onset [27]. Since early treatment is crucial to reducing mortality, developing effective programs to help people respond quickly and obtain medical service is important.

Some studies indicated that age and gender (female) were associated with a delayed presentation for clinical service, and our findings agree with reports that the elderly experienced longer delays in looking for hospital treatment [28,29]. More than 50% of patients over 65 had a late arrival to ER, whereas only 29.0% of younger patients (<65 years) arrived late. This may be due to the elderly having restricted access to medical care or insufficient medical knowledge, particularly when living in rural areas or alone. Furthermore, the elderly may not identify the symptoms of acute coronary disease or recognize their severity. Designing intervention programs to help the elderly recognize symptoms of this disease and provide timely treatment in rural areas requires further research.

This study finds that 65% of female patients delayed more than six hours before being admitted to ER, compared to 34.4% of male patients, concurring with previous studies [28,29]. Females have delayed seeking medical care more than men due to a lower occurrence of acute coronary diseases. Hence, they are more likely to ignore the signs and symptoms. In addition, differences in biological, sociodemographic, and behavioral characteristics between females and males may contribute to the gender discrepancy in time delay. However, since age and gender are not significant predictors for delayed arrival in the multiple logistic model, the relationship between the influence of age, gender, and delayed presentation for clinical service needs further investigation. Therefore, public health authorities should design and propagate programs about early treatment of AMI for the elderly and females.

This study finds that people with more years of formal education have a lower prevalence of delayed presentation to the hospital than those with formal education less than 10 years. It is clear that people with higher educational levels have better knowledge about health status or disease symptoms and how to deal with medical problems. People in the United States with fewer years of education are inclined more have a higher prevalence of cardiovascular disease [30]. The elderly in Kinmen, as well as Taiwan generally, usually have less education than younger people. The actual effect between delayed presentation to the hospital and the education level combined with age needs further investigation.

According to the National Health Insurance Research Database (NHIRD) of the National Health Research Institute in Taiwan, the ratio of NSTEMI to STEMI incidence was 2.47 in 2015 [11]. Still, the prevalence of STEMI is higher than NSTEMI (59.5% vs. 44.5%) in Kinmen, and the reasons for this difference need further investigation. It was found that STEMI was associated with a higher risk of short-term mortality, but NSTEMI was associated with a higher risk of long-term mortality [31]. Early treatment or revascularization is important for better long-term outcomes for both STEMI and NSTEMI, but the late arrival rate to ER did not reach a significant difference of statistics in Kinmen.

We found that Killip Class is a significant issue relating to delayed arrival at ER for medical care of AMI patients in Kinmen. This result is consistent with previous findings from Middle Eastern countries that patients with higher Killip Class sought medical care later than 12 h and those with higher resting heart rate and irregular blood sugar barely showed chest pain at presentation [12]. This has been found to result in physician selection bias where patients with higher Killip Class tend to receive fewer coronary angiography- and evidence-based therapies than patients with Class I. Since high Killip Class was a significant indicator of mortality in STEMI and NSTEMI myocardial infarction and non–ST-elevation acute coronary syndrome, emergency medicine physicians should be attentive to clinical examination in the risk classification for patients with acute coronary conditions.

We performed the binary logistic regression with just two independent variables. The results reveal that age (elderly patients ≥65 years) and higher Killip Class (≥II) are significant factors related to delayed arrival at ER, but the educational level did not reach a significant difference of statistics. Therefore, the relationship between delayed arrival and Killip Class, age, educational level, gender in Kinmen should be investigated in future studies.

### Strengths and Limitations

This study presents the empirical finding that patients’ Killip Class, gender, age, and educational level are important factors related to delayed ER arrival for medical care of AMI patients in Kinmen. Killip Class was found to have the most significant correlation. This information could help provide a direction for future research in planning effective care regimes for AMI patients in areas with insufficient medical resources.

There are some limitations to this study. The sample was taken from the only hospital in Kinmen Island, and the case number was limited, which limited the application of more advanced inferential statistics. Since this is a cross-sectional study, the effects of factors relating to delayed arrival at the ER for medical care need further exploration.

## 5. Conclusions

The study determines that delayed arrival at ER is highly correlated to higher Killip Class. It is important to remind physicians to proceed with risk stratification for patients with acute coronary syndrome. Health authorities should propose effective education programs to the general public, and especially the elderly, for the symptoms and timely treatment of acute myocardial infarction.

## Figures and Tables

**Table 1:** The distribution of sociodemographic and clinical factors among the AMI patients in Kinmen.

	N	%
Gender		
Female	20	17.2
male	96	82.8
Age	Mean ± SD = 63.01 ± 14.53	
≤64	69	59.5
≥65	47	40.5
Marriage status		
Unmarried	6	5.2
Married	90	77.6
Divorced/Separated	11	9.5
Widow	9	7.8
Educational level (years)		
≤9	64	55.2
≥10	52	44.8
Living with family		
Yes	101	87.1
No	15	12.9
Way to ER Visit		
Self	10	8.7
By ambulance	106	91.3
AMI Type		
STEMI	69	59.5
NSTEMI	47	44.4
Killip Classification		
I	60	51.7
II	29	25.0
III	25	21.6
IV	2	1.7
With Hypertension		
Yes	60	51.7
No	56	48.3
With Diabetes Mellitus		
Yes	39	33.6
No	77	66.4
Hyperlipidemia		
Yes	26	22.4
No	90	77.6
Late Arrival		
Yes	46	39.7
No	70	60.3
Total	116	100.0

**Table 2:** The distribution of different factors related to late arrival of AMI patients.

	Late Arrival N (%)			*p* Value
	No	Yes	Total	
Gender *				0.011
Female	7(35.0)	13(65.0)	20(100)	
Male	63(65.6)	33(34.4)	96(100)	
Age **				0.004
≤64	49(71.0)	20(29.0)	69(100)	
≥65	21(44.7)	26(53.3)	47(100)	
Educational level (years) *				0.025
≤9	33(51.6)	31(48.4)	64(100)	
≥10	70(72.9)	26(27.1)	96(100)	
AMI type				0.068
STEMI	24(51.1)	23(48.9)	47(100)	
NSTEMI	46(76.7)	23(33.3)	69(100)	
With Hypertension				0.394
Yes	35(58.3)	25(41.7)	60(100)	
No	35(62.5)	21(37.3)	56(100)	
With Diabetes Mellitus				0.331
Yes	25(64.1)	14(35.9)	39(100)	
No	44(57.9)	33(42.1)	77(100)	
With Hyperlipidemia				0.462
Yes	15(57.7)	11(42.3)	26(100)	
No	55(61.1)	35(38.9)	90(100)	
Killip Classification **				0.001
I	46(76.7)	14(23.3)	60(100)	
≥II	24(42.9)	32(57.1)	56(100)	

*: *p* < 0.05; **: *p* < 0.01.

**Table 3:** The logistic regression analysis of late arrival in different factors. (**a**) The logistic regression analysis of late arrival in age and Killip Classification. (**b**) The logistic regression analysis of late arrival in educational level and Killip Classification.

	OR	95% C.I.	*p* Value
Gender			
Male	Reference		
Female	1.804	0.576–5.648	0.311
Age			
≤64	Reference		
≥65	2.041	0.786–5.298	0.143
Educational Level			
≤9	1.172	0.485–3.338	0.625
≥10	Reference		
Killip Classification			
I	Reference		
≥II	3.616	1.574–8.310	0.002
			R^2^ = 0.224
(**a**)			
Age			
≤64	Reference		
≥65	2.602	1.149–5.895	0.022
Killip Classification			
I	Reference		
≥II	3.936	1.736–8.823	0.001
			R^2^ = 0.209
(**b**)			
Educational Level			
≤9	2.060	0.908–4.643	0.084
≥10	Reference		
Killip Classification			
I	Reference		
≥II	4.214	1.835–9.266	0.001
			R^2^ = 0.186
